# The effects of message framing on US police chiefs’ support for interventions for opioid use disorder: a randomized survey experiment

**DOI:** 10.1186/s40352-024-00306-4

**Published:** 2024-12-19

**Authors:** Brandon del Pozo, Saba Rouhani, Amelia Bailey, M. H. Clark, Kaitlin F. Martins, Fatema Z. Ahmed, Danielle Atkins, Barbara Andraka-Christou

**Affiliations:** 1 The Warren Alpert Medical School of Brown University, Providence, RI United States; 2 Rhode Island Hospital, Brown University Health, Providence, RI United States; 3 New York University School of Global Public Health, New York, NY United States; 4 Brown University School of Public Health, Providence, RI United States; 5 University of Central Florida College of Community Education and Innovation, Orlando, FL United States; 6 Florida State University Askew School of Public Administration and Policy, Tallahassee, FL United States

**Keywords:** Harm reduction, Public health, Policing, Police, Law enforcement, Communication, Framing, Overdose prevention, Safe injection, Syringe service programs, Good Samaritan laws, Naloxone

## Abstract

**Background:**

US chiefs of police hold significant influence over the perceived acceptability and appropriateness of interventions for opioid use disorder (OUD) among the public, elected officials, and subordinate officers. This study assessed whether police chiefs’ support for such interventions was sensitive to framing an intervention’s benefits in terms that emphasize public health and harm reduction outcomes, versus terms typically indicative of public safety outcomes.

**Methods:**

A two-armed survey utilizing a randomized, between-subjects design tested framing-based variance in support among US chiefs of police for overdose prevention centers, syringe service programs (SSPs), Good Samaritan laws, police naloxone distribution, trustworthiness of officers in recovery from OUD, and related propositions. Of 1,200 invitations, 276 chiefs participated (23%). The two experimental arms (*n* = 133, *n* = 143) were demographically balanced between both each other and non-respondents.

**Results:**

Chiefs were more likely to agree that their mission was protecting public safety than protecting public health, even when both were defined using public health outcomes. Chiefs expressed significantly greater support for “overdose prevention sites” than “safe injection sites” (*p* = .018), low levels of support for SSPs regardless of framing (18% safety; 19% health), and comparably more support for Good Samaritan laws based on framing (62% safety vs. 54% health). Respondents voiced low levels of trust in officers recovering from OUD generally (31%), and significantly lower levels of trust when recovery involved the medication buprenorphine (10%; *p* < .001). Senior chiefs were significantly more likely to support SSPs (aOR 1.05; CI 1.01, 1.09) and overdose prevention sites (aOR 2.45; CI 1.13, 5.28) than less senior chiefs.

**Conclusions:**

In this cross-sectional survey experiment, support for some interventions for OUD was greater among US chiefs of police when framed to emphasize positive public safety outcomes. Research is required to better understand low support for SSPs, mistrust of officers in recovery for OUD, and greater support for OUD interventions among senior chiefs.

## Introduction

There were over 109,000 drug-related overdose deaths in the US in 2022, with most attributed to opioids (Ahmad 2023). Harm reduction strategies administered through syringe service programs (SSPs) and medications for opioid use disorder (MOUD) provide evidence-based interventions to reduce this unprecedented overdose mortality (Fernandes et al. 2017; NASEM 2019). However, few individuals with opioid use disorder (OUD) receive MOUD (Jones et al. 2023; Volkow and Blanco 2023), and there is growing recogntion that policing interferes with linkages to treatment and harm reduction. For example, police responses to overdose incidents often lead to arrest rather than connection to treatment (Ray et al. 2022), and drug seizures have been found to increase fatal overdoses among people who use drugs (PWUD) (Ray et al. 2023). A police commitment to discretionary behaviors that link PWUD to effective public health interventions and minimize the negative health outcomes of enforcement is critical for reducing rates of fatal overdose and the sequelae of problematic substance use (del Pozo et al. 2023; del Pozo, Sightes, et al. 2021; del Pozo et al. 2021).

Several factors work against such a commitment, however. Principal among them is stigma toward PWUD (Reichert, del Pozo, et al. 2023), where younger officers, those with a lower education level, and those who believed addiction is a moral failing more likely to stigmatize PWUD and to offer less support for public health interventions for problematic substance use (Kruis et al. 2020; Kruis et al. 2021; Murphy and Russell 2021). Stigma against PWUD is not unique to police, as it is highly prevalent among the general population (Jalali et al. 2020; Volkow 2020), where it is associated with support for punitive responses to substance use (Kennedy-Hendricks et al. 2017). Addressing police stigma is critical, however, because officers frequently interact with PWUD (Goulka et al. 2021) and play a critical role in the decision to utilize these touchpoints to promote public health interventions (Beletsky et al. 2015; Wagner et al. 2015).

Research has found that police feel they have a role in overdose response (Carroll et al. 2020; Filteau et al. 2022; Lloyd et al. 2023; Ray et al. 2015), reporting it as instrumental to their public safety mission (Green et al. 2013; Reichert et al. 2023). At the same time, overdose response is often in tension with officers’ perceived legal duties, such as executing arrest warrants seizing contraband, or maintaining public order (Lloyd et al. 2023). Insofar as these tensions place fundamental lifesaving duties in conflict with legal ones, they call for police to reconcile their priorities (del Pozo 2022). Similar concerns exist around overdose prevention centers (OPCs), where individuals can use drugs under the supervision of trained staff. OPCs have low levels of public support in the United States, often out of a concern that they promote crime and disorder, despite initial findings that the first two officially sanctioned ones in the United States have not done so (Chalfin et al. 2023). How police leaders evaluate these concerns has the potential to shape the public’s acceptance of additional centers.

In this way, municipal chiefs of police have the power to influence opinion, both within their communities and their agencies (Wurcel et al. 2023), where police officers have significant discretion in their enforcement decisions about drug-related incidents (del Pozo, Reichert, et al. 2023), and the attitudes and beliefs of mid-level supervisors can in turn shape employee behavior toward public health and harm-reduction initiatives (Marotta et al. 2023; del Pozo, Goulka, et al. 2021; del Pozo, Sightes, et al. 2021). Additionally, if police chiefs have supportive views of public health interventions for problematic substance use, they may discourage officers from interfering with the work of harm reduction providers in the field, or use their command authority to de-prioritize police presence and enforcement around SSPs and OPCs, promoting their undeterred utilization. Efforts to increase support for effective interventions for OUD may therefore require constructively engaging chiefs of police, and such efforts would benefit from evidence about effective communication strategies. While advocates differ in their opinions about whether police should have a role in harm reduction themselves (e.g., deploying and distributing naloxone), police attitudes will matter regardless: they profoundly shape the local policy environments that make community harm reduction programs possible.

In seeking to shore up support for public health responses to OUD among chiefs of police, research suggests the communication strategy of “framing” can play a role in shaping support for public health interventions. Specifically, people are more likely to support an intervention when it is accurately framed using goals they inherently value, such as preventing death. For example, research suggests US adults are significantly more supportive of “overdose prevention sites” than “safe consumption sites,” despite being described as offering identical services (Barry et al. 2018; Socia et al. 2021). In this example, a “consequence frame” foregrounds the repercussions of failing to prevent overdose. In contrast, the phrase “safe consumption” more readily evokes recreational substance use, a highly stigmatized activity (Barry et al. 2018). In this vein, experienced harm reduction advocates in one study felt the most persuasive messaging strategies for promoting their initiatives “align with audience-specific values,” an approach they specifically recommended for police, who they felt should be addressed using public safety framing (White et al. 2023).

This study aims to provide insights for more effective communication with chiefs of police by assessing the extent to which their attitudes and beliefs about interventions for OUD are sensitive to framing. We hypothesized that framing harm reduction and public health interventions as promoting public safety ends (such as reducing death and injury) would generate more support among chiefs than emphasizing health outcomes. Our hypothesis was based on research that the general public continues to see harm reduction as controversial (Novotna 2023), and the premise that police chiefs are more likely to conceptualize their primary responsibility as protecting safety rather than health. By this logic, when an intervention for OUD is phrased with a principal focus on public safety outcomes versus its harm reduction or public health evidence base, it may reduce the chiefs’ perceptions of a tension between public safety and public health approaches to the problem. To test our hypothesis, we used an experimental survey.

## Methods

### Experimental design

This cross-sectional survey experiment employed a randomized, controlled, between-subjects design. It used a master sample of 1,200 randomly selected US chiefs of police to construct two experimental arms of 600 randomly-assigned chiefs. The study was part of a larger project that examined police chiefs’ attitudes toward topics relevant to substance use disorder, and their perceptions of changes in policing since the murder of George Floyd. Apart from the account of the two experimental arms utilized here, our methods were presented in detail in an earlier publication were presented in detail in an earlier publication (del Pozo, Rouhani, et al. 2024).

### Population and sampling

**Fig. 1:**
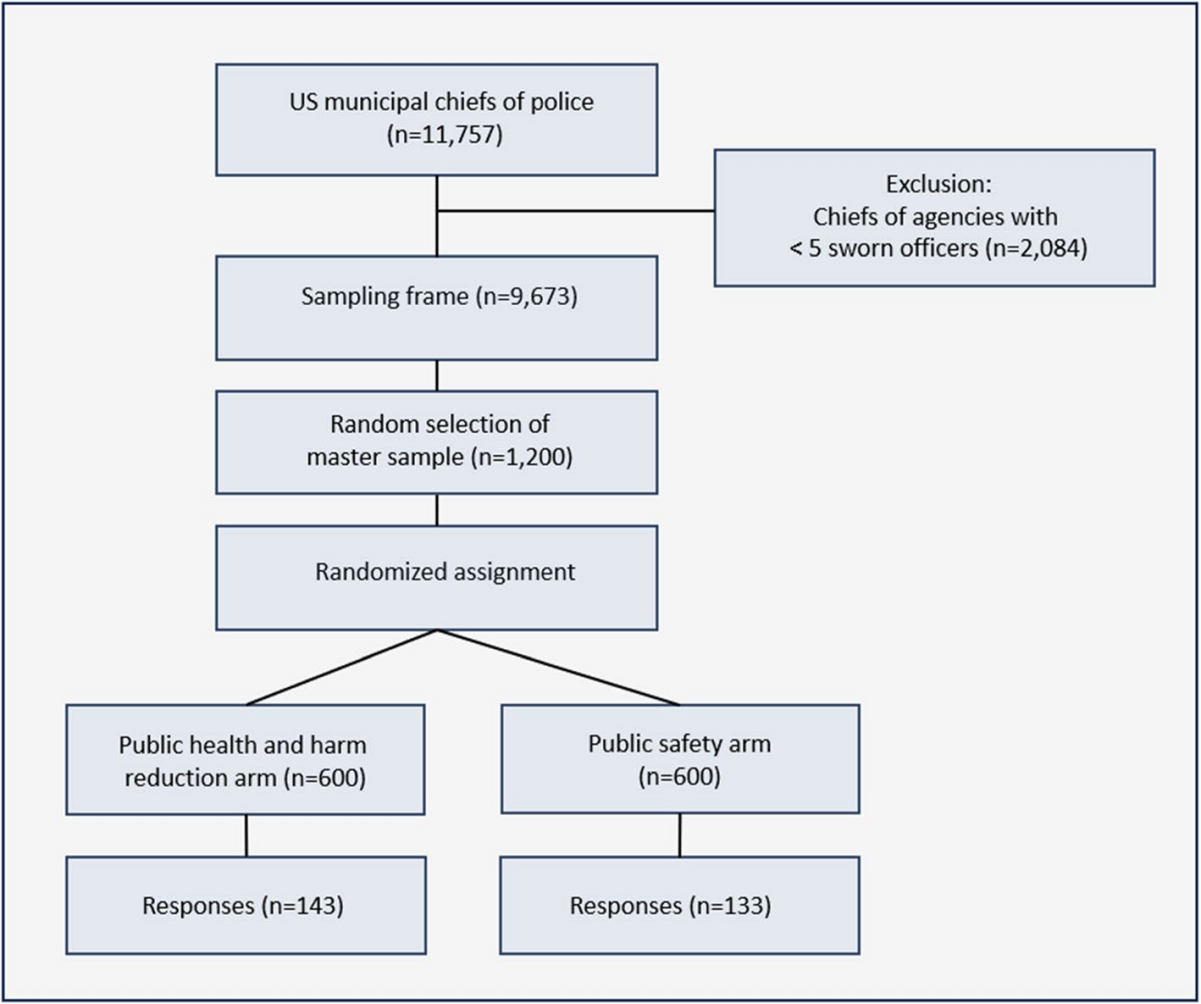
Experimental design: sampling and assignment

Our sampling frame for random selection was drawn from the records of a vendor that maintains demographic data and contact information for the nation’s police agencies, including their executive leadership (NPSIB, 2022). We first excluded the heads of state police agencies, sheriff’s departments, or departments such as park, university, or railroad police, owing to their heterogenous duties and responsibilities toward PWUD. For example, sheriffs typically administer jails in addition to their policing duties, leaving open the possibility that their views could be positively or negatively shaped by their unique responsibility for the health, welfare, and control of an incarcerated population. As illustrated in Fig. 1, from the remaining 11,757 municipal police departments, agencies with fewer than five sworn officers (*n* = 2,084) were excluded. This restricted our sample to larger police departments under the rationale they were more likely to be contending with the policy issues examined by our instrument. The remaining 9,673 departments were assigned random numbers and placed in ascending order. The first 1,200 agencies were drawn into our master sample, which consisted of 12% of the nation’s municipal police departments with five or more officers. Since the sample had been randomly drawn and ordered, randomized assignment to the two experimental arms consisted of dividing the list in half at the 600th row. The first group was assigned the public health instrument, and the second group was assigned the safety instrument.

### Survey instrument

We created two versions of a 37-item instrument, where nine of the items employed alternate framings for the purposes of this experiment, one hereafter broadly referred to as the “health” arm and the other the “safety” arm due to the principal focus of each item, such as an emphasis on harm and health vs. death and injury, the centering of police vs. health or harm reduction actors, or a focus on an evidence-based public health practice rather than a more general one. The experimental items included propositions about SSPs, OPCs, Good Samaritan laws meant to reduce incidence of arrest at the scene of an overdose, police naloxone distribution, strategic emphases on public safety vs. public health, and support for officers in recovery from OUD. The specific phrasing used for the items in each arm of the experiment is presented in Table 1. Respondents were asked to rate the extent of their agreement or disagreement using a sematic differential scale (i.e., a 1–7 visual analog scale with anchoring language). In addition to these propositions of interest, we also collected demographic data, allowing us to assess homogeneity between the two arms, and therefore how confident we could be that variance in responses between arms arose from the effects of framing.

**Table 1:**
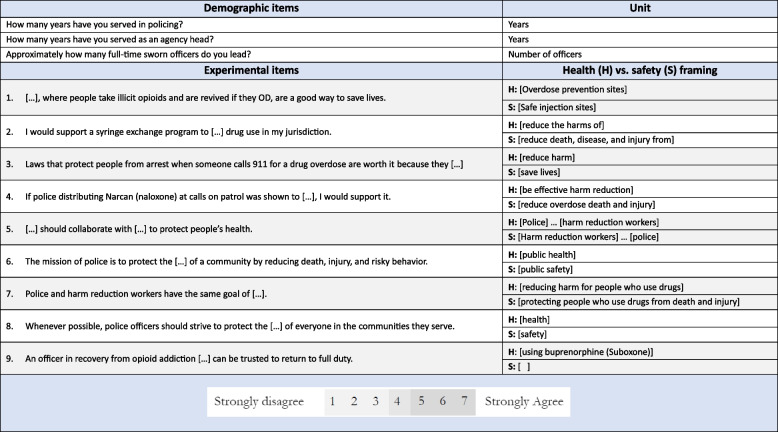
Items and their alterative phrasing for health and safety framing

In designing our instrument, we considered that a response bias might arise from chiefs more averse to public health and harm reduction being less inclined to return the survey regardless of how our items were framed, skewing our respondents toward a group more amenable to these approaches in the first place. To reduce this possibility, the first nine items of the survey were about the effects of the murder of George Floyd on police recruitment, retention, morale, and the perceived risks of proactive policing, while the last three items were about attempts at defunding their departments and resulting changes in community safety. We hypothesized these questions would be of interest to chiefs regardless of their views about public health and harm reduction, and would motivate them to complete and return the survey. An analysis of these items is presented in del Pozo, Rouhani, et al. (2024).

### Data collection

The first wave of surveys was mailed to each chief of police in January of 2022. Each letter contained a stamped return envelope with a unique identifier to populate a second wave of mailings based on nonresponse. After about six weeks, the second wave of surveys was mailed. The data of respondents were then entered and cleaned, and the zip code of each responding chief’s headquarters was used to link their record to a designation of urban (metropolitan or micropolitan) or rural (small town or rural), the size of their agency, the size of the population it served, and its US Census Bureau region. The records were then stripped of identifiers, anonymizing the sample. The cost per response in this study (e.g., list procurement, printing, and postage, but excluding research effort) was approximately $18.57.

### Data screening and cleaning

When respondents circled two consecutive numbers or circled the space between two numbers but neither number itself, we entered their average response value. The handful of cases where a respondent wrote “same” or “did not affect” were coded as 4, the neutral value on the scale. We chose not to eliminate these responses because they reflected the intention to provide our team with data, and we were unambiguously able to understand the intention. Indecipherable responses, in contrast, were coded as missing data, as were omissions. In two cases where a person had left their response blank, prior to anonymization, publicly available data were used to determine how long they had served as a chief of police. Visual inspection did not suggest a pattern to any of these data points. All alterations were noted in the dataset.

### Analysis

An analysis to detect significant nonresponse bias was conducted using the demographic covariates of respondents and nonrespondents. Among respondents, we additionally compared covariate data between the experimental arms to assess whether the groups were appropriately balanced. For these analyses, we utilized chi-square statistics (categorical) and two-sided t-tests (continuous) to describe bivariate differences in responses between groups. These results are provided in Sect. 3.1.

To analyze respondent data, we grouped responses into three dispositions toward each proposition: disagreement (1–3), neutrality (4), and agreement (5–7). We then utilized chi-square statistics to assess the significance of differences in response between framings. We used ordinal logistic regression to estimate the effect of public safety framing, as compared to public health framing, on each of our nine randomized survey items. Ordinal logistic regression is a model for ordered categorical data; resulting odds ratios can be interpreted as the relative odds of endorsing a higher-level of support for a survey item as compared to the immediately lower level of support as a result of receiving "public safety" framing. In other words, odds ratios summarize the effect of switching from health framing to safety framing on moving from disagree to neutral, and on moving from neutral to agree. Models were adjusted for agency size, region, urbanicity, respondent tenure, and population size.

We also tested the consistency of our findings across seven-point and three-point measurement scales, as we grouped the original seven-point Likert-type responses into three ordinal categories (agree, disagree, neutral). To do so, we used a Wilcoxon rank sum test of the median responses, which tested whether the distribution in one of the experimental arms is higher (i.e., more “in agreement”) than in the other arm by comparing the number of responses that fall above the global median in each group. We conducted an analysis of missing item responses to to determine if they were missing completely at random by comparing distributions of observed characteristics between respondents with and without missing values on other traits. Finally, a chi-square test was used to assess the probabiltiy that an imbalance in the observed number of responses for each arm was not due to chance. All analyses were conducted in Stata v.17 (StataCorp. 2021).

### Ethics

As it consisted of the administration of a survey that did not exceed minimal risk, our research was deemed exempt by Rhode Island Hospital’s IRB, with a waiver of written consent, and was designed and conducted in accordance with the Common Rule. All survey respondents received an Explanation of Research prior to data collection.

## Results

### Respondent characteristics

Of the invitations to participate mailed over two waves, 23% of chiefs (*n* = 276) completed the survey: 143 in the health arm, and 133 in the safety arm. This response rate compared favorably to that of other published survey studies of chiefs of police, a point taken up in the Discussion. Respondent characteristics are presented in Table 2. Wisconsin, Illinois, Indiana, Michigan, Pennsylvania, Texas, Ohio, and Indiana produced more than 10 responses; the other states produced fewer. Chiefs in all states were sampled, and at least one from each state responded, except for Hawaii, Wyoming, South Dakota, Vermont, and Nevada.

**Table 2:**
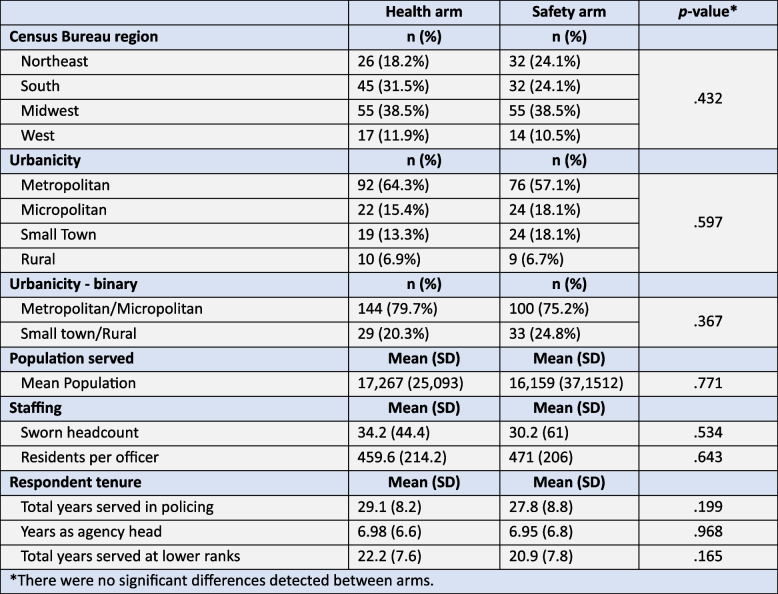
Respondent characteristics, *n* = 276

The largest participating agencies had nearly 500 sworn officers serving populations approaching 300,000; mean agency size was 32.3 (*SD* = 53.0), and mean population served was 16,733 (*SD* = 31,431). The chiefs who returned the survey led police departments collectively serving 4.6 million US residents. Respondents led agencies in the Northeast (21%), South (28%) Midwest (40%), and West (11%). The majority (61%) led agencies in metropolitan areas. Respondents had served a mean of seven years as the head of their agencies, with a mean 28.4 years of service in policing in total. There were no significant demographic differences in response to the survey, except by region: chiefs in the South were significantly less likely to return the survey than chiefs in the Midwest (19% vs. 29%; *p* = 0.002). See del Pozo, Rouhani, et al. (2024) for an exposition of these analyses. The *p* values in Table 2 here demonstrate that the health and safety arms were balanced on demographic covariates, i.e., that random assignment was effective in controlling selection bias.

### Analyses of experimental items

Complete response analyses by item can be found in Table 3. When a place “where people take illicit opioids and are revived if they overdose” was described as “a safe injection site,” 10% (*n* = 11) of the chiefs assigned to the safety arm agreed it was a “good way to save lives.” When it was instead described as an “overdose prevention site,” 22% (*n* = 28) of the chiefs assigned to the health arm agreed. The difference in distribution of the rankings of agreement levels was significant (*p* = 0.018).

**Table 3:**
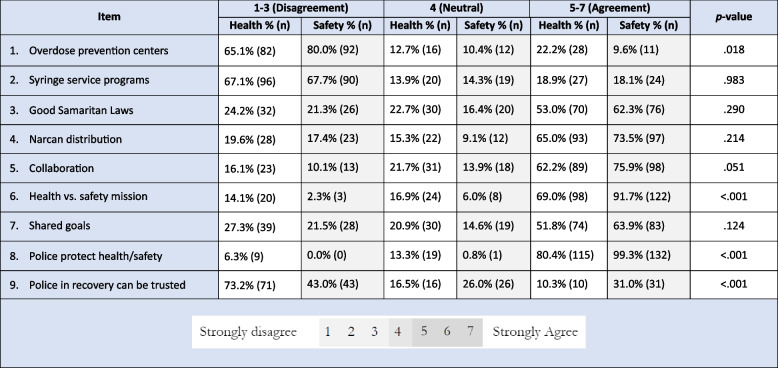
Responses by experimental item, *n* = 276

In contrast, SSPs received low levels of support from chiefs, regardless of whether their value was cast as reducing the harms of drug use, or reducing death, disease, and injury: respectively, only 19% in the health arm and 18% in the safety arm expressed agreement they would support SSPs in the pursuit of such ends (*p* = 0.983).

Good Samaritan laws garnered comparatively more support than SSPs: 53% (*n* = 70) of chiefs in the health arm agreed they were “worth it” because they “reduce harm,” and 62% (*n* = 76) in the safety arm agreed because they “save lives.” In both the health and safety arms, under a quarter of chiefs disagreed they were worth it under either rationale: 24% (*n* = 32) and 21% (*n* = 26) respectively. Chiefs of police were more likely to support their officers distributing the overdose reversal medication Narcan (naloxone) on patrol if it was “shown to reduce overdose death and injury” (74%, *n* = 97) rather than “reduce harm” (65%, *n* = 93). Neither difference between arms was significant.

When asked if “the mission of police is to protect the public health of a community by reducing death, injury, and risky behavior,” 69% (*n* = 98) of chiefs in the health arm agreed. When asked if reducing the same outcomes reflected a mission to protect the public safety of a community, 92% (*n* = 122) in the corresponding arm agreed, a significant difference in the distribution of the rankings of agreement levels (*p* < 0.001). This significance was also reflected in a more basic distinction. When the arms were asked if “whenever possible, police officers should strive to protect the [health/safety] of everyone in the communities they serve,” 80% (*n* = 115) of the health arm agreed, compared to 99% of the safety arm (*n* = 132), a significant difference (*p* < 0.001).

Between the study arms, chiefs of police were more likely to agree that “police and harm reduction workers have the same goal of protecting people who use drugs from death and injury” (64%, *n* = 83) rather than “reducing harm for people who use drugs” (52%, *n* = 74) (*p* = 0.124). Chiefs were more likely to agree that harm reduction workers should collaborate with police to protect people’s health (76%, *n* = 98) than to agree police should collaborate with harm reduction workers to the same end (62%, *n* = 89) (*p* = 0.051), suggesting that chiefs were more supportive of collaboration when it centered on their own agencies and their public health and harm reduction counterparts bore the onus of the collaboration.

Chiefs expressed limited trust in officers in recovery for OUD. In the safety arm, where the term “recovery” was used generally, 69% (*n*= 69) were neutral or disagreed that an officer in recovery could be trusted to return to full duty. When officers in the health arm were additionally told recovery consisted of using buprenorphine, an item that we included in that arm because it reflects the best available evidence about effective treatment for OUD (Wakeman et al. 2020), neutrality (17%) and disagreement (73%) significantly rose to a collective 90%, reflecting a significant difference in the distribution of the rankings of agreement levels (*n* = 87; *p* < 0.001). The rate of omission for this item was also uncharacteristic: 46 in the health arm (32%), and 33 in the safety arm (25%). The mode omission rate for the other items across arms was 0%, and the mean was 3%.

### Adjusted odds ratios and demographic associations

Table 4 expresses the relative support for key policy items by arm as adjusted odds ratios. Odds of support for OPCs were significantly higher among chiefs asked to consider them as overdose prevention sites versus safe injection sites (aOR = 2.45; CI = 1.13, 5.28; *p* < 0.05). After adjusting for study arm and other covariates of interest, the odds of supporting SSPs were significantly greater among chiefs who had served in the profession for longer (aOR = 1.05; CI = 1.01, 1.09; *p* < 0.05) and the odds of support for GSL were significantly reduced in non-urban settings (aOR 0.46; CI = 0.24, 0.87; *p* < 0.05). Time in service was also associated with reduced odds of support for patrol officers taking on the additional duty of naloxone distribution (aOR = 0.97; CI = 0.94, 1.00; *p* < 0.1). No other differences were found at the *p* < 0.05 level of significance.

**Table 4:**
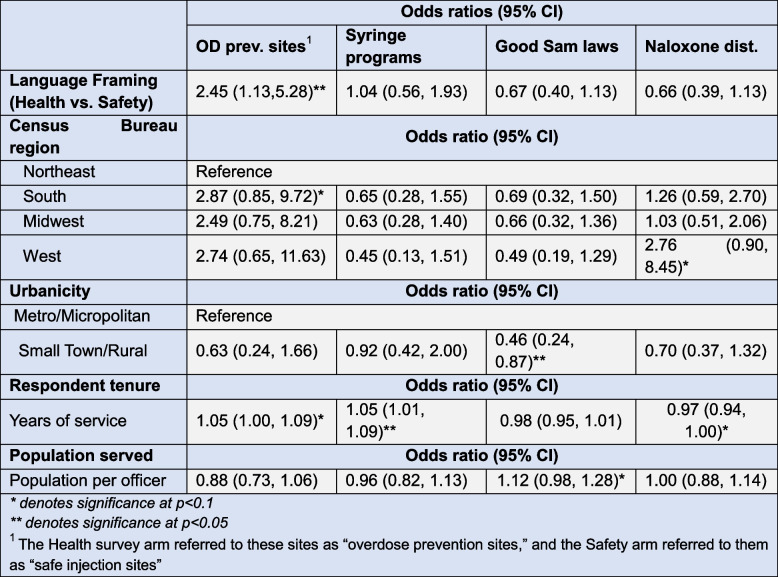
Odds of police executive support of public health and harm reduction interventions by language framing and covariates of interest, *n* = 276

### Additional analyses

Using a Wilcoxon rank sum test with the seven-point rating scales provided in the instrument, we found that the overall endorsement of statements related to the police mission, collaboration with harm reduction workers, and Good Samaritan laws was higher when phrased in terms related to safety than health, which would further affirm our hypothesis. However, when analyzed this way, the difference in support for “overdose prevention sites” compared to “safe injection sites” was no longer statically significant, possibly arising from that fact that although there was a difference between the groups, overall support remained low between them, and this analysis tested against mean ranks.

We also tested two additional possibilities: that there was a significant association between missing responses for a given item and any of the demographic variables in the survey, and that the difference in number of responses for each arm (133 of 600 for safety (22.2%), and 143 of 600 for health (23.8%)) was unlikely to be due to chance. In the former case, none of the associations were significant (i.e., all values were missing completely at random). Regarding differences in response rates between the two arms, our analysis found the difference was likely due to chance (*p* = 0.55).

## Discussion

This study is the first we are aware of to use an experimental survey to examine effects of framing on support for OUD interventions among a randomly selected and assigned sample of US chiefs of police. The results confirm beliefs among harm reduction and public health advocates that framing matters when engaging police officials for support (White et al. 2023). Differences in responses among chiefs reflected a consistent preference for interventions described as benefitting public safety versus public health, and in some cases those differences were significant. Item 6 revealed police chiefs’ relative preference for conceiving of their mission as the production of public safety, even when it was defined using traditional public health concepts, i.e., reducing morbidity, mortality, and the underlying risk behaviors at the community (i.e., population) level (Rose 1985).

Although chiefs expressed significantly greater support for “overdose prevention sites” as compared to “safe injection sites” (Barry et al. 2018; Socia et al. 2021), overall support for the intervention remained low, which tracks opinions among the public at large (McGinty et al. 2018). While initial results indicate the officially sanctioned centers in New York City have had no discernible negative impact on public safety (Chalfin et al. 2023), US police officials have had little exposure to these centers in practice. Efforts to open new centers in Minnesota and Rhode Island may allow chiefs to witness the sites operating in other settings, so it is possible their opposition to OPCs will decrease if the centers continue to demonstrate few negative public safety outcomes.

Chiefs of police voiced low levels of support for SSPs regardless of how they were framed. This finding is in tension with research that concludes educating officers about the positive impacts of SSPs on police occupational safety (e.g., a lower likelihood of infectious needlestick injuries), can significantly improve police attitudes toward SSPs (Baker et al. 2022; SHIELD 2021; Strathdee et al. 2015). It is possible that the short and direct (i.e., one sentence) public safety framing utilized in this study was inadequate for changing chiefs’ attitudes about SSPs, but a more detailed and thorough framing might do so.

The police chiefs sampled were, on balance, hesitant to trust an officer in recovery for OUD, but were particularly distrusting of officers in treatment with the partial agonist opioid buprenorphine; it is possible that chiefs in the study arm that did not mention buprenorphine assumed recovery entailed an abstinence-only approach. Negative perceptions of people in treatment for OUD with buprenorphine are not unique to policing, however: some state medical and nursing boards prohibit clinicians from returning to work while undergoing buprenorphine treatment (Beletsky et al. 2019). Negative perceptions of buprenorphine are especially problematic given that it significantly reduces risk of overdose (Sordo 2017), is an effective form of MOUD (Wakeman et al. 2020), and has a demonstrated history of playing almost no role in recreational misuse (Chilcoat et al. 2019) or overdose (del Pozo, Atkins, et al. 2023). Such stigma could hinder police officers from utilizing a lifesaving treatment intervention.

Police chiefs in our study expressed the belief that their profession’s role was fundamentally similar to that of harm reduction, but were more likely to agree that harm reductionists should collaborate with police than that police should collaborate with harm reductionists. Centering one’s own profession in collaboration is likely a common trait among organizational leaders. In this case, however, it may make collaboration between police and harm reduction professionals challenging given the considerable power imbalance between them, especially in terms of influence over public policy decisions. Given this inherent imbalance, police leaders and other government officials may need to deliberately ensure harm reductionists and public health actors have adequate voice and standing during collaborative efforts.

The positive, significant association between the length of time a respondent had served in policing and their support for evidence-based OUD interventions such as SSPs and OPCs has the potential to be instructive. Our finding aligns with that of a prior study that found officer tenure was positively associated with supportive attitudes toward public health interventions for PWUD (Rouhani et al. 2019). Perhaps the longer chiefs have held their offices, the more exposure they have had to the ineffectiveness of status quo responses to the overdose crisis. If that is the case, supportive senior chiefs could be leveraged to convey their rationale to less senior leaders. Likewise, future research should determine if increased levels of exposure to incidence of problematic substance use and overdose associate with increased support for public health and harm reduction interventions even after controlling for chiefs’ tenure. Conversely, the lower odds of support among more senior chiefs for officers distributing naloxone to the community while on patrol may reflect a hesitance to further expand police duties, a phenomenon tenured chiefs have witnessed over the past several decades for societal problems such as mental health crises (Watson and El-Sabawi 2023).

In the pursuit of effective municipal responses to addiction and overdose, our results have actionable policy and communication implications. In describing OUD interventions to police leadership using a public safety vernacular, advocates may be more likely to garner their support by tying interventions to the police chief’s perceived professional mission. Relatedly, community advocates could emphasize to police chiefs that OUD interventions have both public safety and public health implications, framing them as complementary pursuits. In the short term, the findings indicate messaging for chiefs of police that focuses on the discrete safety implications of public health and harm reduction strategies. Mid-term advocacy efforts would convey that these measures are, in most cases, congruent and operationally compatible with public safety goals, while encouraging police leaders to assess the health as well as public safety outcomes of their work. The long-term objective would to be to pursue municipal public health and public safety in tandem, with an emphasis on ensuring one does not displace the other to a degree the residents of a municipality–and especially their most vulnerable communities—cannot accept.

### Limitations

This study has limitations. It sampled chiefs from police departments with five or more officers, but it did not oversample the comparatively few leaders of the large departments wield great influence over the US urban population, nor did it sample sheriffs, the elected officials who perform the dual roles of patrol and jail administration in over 3,000 of the nation’s counties, many of them rural and not served by municipal agencies. Future research should do so. The overall sample also captured significantly fewer respondents from the Southern Census Bureau region, limiting our ability to generalize about the police leaders there. However, our response rate of 23% is typical of police survey research (Nix et al., 2019). A Police Executive Research Forum survey of its national membership of 1,068 law enforcement executives about staffing challenges in policing resulted in 266 completed questionnaires, a response rate of 25% (PERF 2023). Elsewhere, a survey about Oregon police executives’ receptivity to research yielded a 26% (*n* = 45) response rate (Telep & Winegar, 2015), comparable to a contemporary study that surveyed chiefs in 340 agencies reporting a line of duty cardiac death from 1984–2010, producing a 27% (*n* = 93) response rate (Korre et al., 2014). Finally, a survey about crisis intervention training targeting 746 Georgia police chiefs and sheriffs yielded a response rate of 27% (*n* = 204) (Compton et al., 2015). This study had a response rate comparable to those of previously published research surveying chiefs of police, with a result that may better represent the US population of municipal chiefs considering its sample size and random sampling approach.

Missing responses may also pose a limitation if social desirability, other biases, or perceived professional liabilities led to chiefs not to respond with levels of strong agreement or disagreement that would have altered the magnitude or significance of the findings. It is possible, for example, that despite assurances of anonymity, chiefs declined to respond to statements assessing their support for officers in recovery for OUD because it would indicate their official policy stance on the matter, and subject them to scrutiny or litigation in actual incidents. It may also be possible that chiefs had strong negative feelings about harm reduction interventions but were concerned about disclosing them to researchers. Our analyses did not find missingness was significantly associated with any of demographic characteristics we captured, however, so if the missing data has left extreme responses undetected, such views among chiefs were not associated with several variables that could have explained it. Likewise, while there was a difference in the response rate between arms, our analysis suggested it was likely due to chance.

For these reasons, we did not conduct sensitivity analyses that assigned extreme values to missing responses, or assigned them to synthetic safety arm responses that would balance the two groups. Few respondents answered with extreme values, and doing so would have suggested missingness had a greater effect than would have prevailed if we had collected a larger or more complete sample. Nonetheless, this discussion points to a limitation of our findings that is worth noting. We also advise caution in interpreting our findings in the context of the urbanicity data we provide. It is possible for chiefs of police of small towns and sparsely populated areas to nonetheless be categorized as leading a department located in a larger metropolitan or micropolitan area. Although these classifications did not reveal significant differences in response apart from support for Good Samaritan laws, the ambiguity inherent in them can reduce the clarity of our findings.

## Conclusion

In the continued pursuit of evidence-based public health responses to the opioid crisis, securing the support of police officials can be a strategy of reluctant necessity, or an opportunity to form more productive partnerships between stakeholders. In either case, emphasizing the public safety benefits of effective interventions may constitute a more persuasive communication strategy than relying on the merits of improved health outcomes alone, however sufficient they may be in principle. Police chiefs heavily influence the perceived appropriateness and acceptability of municipal public health strategies, and while our study found negative beliefs about these strategies in a national sample of chiefs, it also provides evidence that stressing the potential for public health interventions to promote public safety might help reduce them.

The need to be persuasive about the public safety benefits of public health interventions remains acute. For example, Oregon’s decriminalization of personal drug possession foundered, in part, for its portrayal as having unacceptable consequences for public safety (del (del 2024; Kim 2024; Smiley-McDonald et al. 2023), and New York City’s overdose prevention centers have yet to be emulated elsewhere owing to such concerns. Significantly more research is needed into effective ways to persuade police chiefs and others who yield substantial political power about the benefits of effective public health interventions for PWUD. Perhaps most revealing, however, is that the language used in this study that was most evocative of public safety referenced the reduction of death and injury. These are critical health outcomes as well, suggesting chiefs of police, public health officials, and harm reductionists are all in the business of public health and in pursuit of shared goals. This mutual commitment ought to provide common ground, so that there is such enduring disagreement about the best path forward should be a source of concern and introspection for everyone who claims to take this commitment seriously.

## Data Availability

Data will be made available to researchers upon review and approval of the authors, for purposes broadly related to the aims of its original collection, without investigator support.
